# Auxosporulation in *Paralia guyana* MacGillivary (Bacillariophyta) and Possible New Insights into the Habit of the Earliest Diatoms

**DOI:** 10.1371/journal.pone.0141150

**Published:** 2015-10-20

**Authors:** Irena Kaczmarska, James M. Ehrman

**Affiliations:** 1 Biology Department, Mount Allison University, 63B York Street, Sackville, New Brunswick, Canada; 2 Digital Microscopy Facility, Mount Allison University, 63B York Street, Sackville, New Brunswick, Canada; CSIR- National Institute of Oceanography, INDIA

## Abstract

**Background:**

Diatoms are one of the most ecologically important aquatic micro-eukaryotes. As a group unambiguously recognized as diatoms, they seem to have appeared relatively recently with a limited record of putative remains from oldest sediments. In contrast, molecular clock estimates for the earliest possible emergence of diatoms suggest a considerably older date. Depending on the analysis, *Paralia* and *Leptocylindrus* have been recovered within the basal molecular divergences of diatoms. Thus these genera may be in the position to inform on characters that the earliest diatoms possessed.

**Findings:**

Here we present auxospore development and structure of initial and post-auxospore cells in a representative of the ancient non-polar centric genus *Paralia*. Their initial frustules showed unusual, but not unprecedented, spore-like morphology. Similarly, initial frustules of *Leptocylindrus* have been long considered resting spores and a unique peculiarity of this genus. However, even though spore-like in appearance, initial cells of *Paralia* readily resumed mitotic divisions. In addition, *Paralia* post-auxospore cells underwent several rounds of mitoses in a multi-step process of building a typical, “perfect” vegetative valve. This degree of heteromorphy immediately post-auxosporulation is thus far unknown among the diatoms.

**Implications:**

A spore-related origin of diatoms has already been considered, most recently in the form of the “multiplate diploid cyst” hypothesis. Our discovery that the initial cells in some of the most ancient diatom lineages are structurally spore-like is consistent with that hypothesis because the earliest diatoms may be expected to look somewhat similar to their ancestors. We speculate that because the earliest diatoms may have appeared less diatom-like and more spore-like, they could have gone unrecognized as such in the Triassic/Jurassic sediments. If correct, diatoms may indeed be much older than the fossil record indicates, and possibly more in line with some molecular clock predictions.

## Introduction

Diatom life history consists of two phases. Vegetative propagation multiplies existing genotypes as long as the local environment supports their growth, while sexual reproduction generates new gene combinations for future environmental opportunities [1]. Thus this vegetative stage may consist of an uncountable number of individual diploid cells, all descendents of a single zygote, propagated over the course of many mitoses over a number of years, in some species [2]. The sexual part of the life history is comparatively short, generally lasting a few days [2]. Typically it engages a considerably smaller number of sexually competent cells, which are restricted to those in a species-specific cell-size range [2]. Sexually competent cells may sexualize if the local environment issues a set of species-specific clues [1]. Unlike plants and other algae, following meiotic gametogenesis (with no intervening mitoses), and successful syngamy, a diploid initial progeny cell is produced. Each diatom initial cell begins a round of mitoses, propagating its own, specific genetic makeup as a clonal cell-line (or cohort) of individuals. How the morphology of the cell walls in one such cell-line is shaped by the temporal and spatial interaction of nature (genetics) and nurture (optimal vs. tolerable environment, etc.) over the life-span of one specific genotype (including cell-size diminution) is virtually unknown.

Diplontic life histories are infrequent among algae. Post sexual mitotic propagation in diatoms leads to a theoretically immortal clonal cohort of separate diploid cells dispersed throughout the environment. It is the first stage of diatom life history, the mitotically derived individual cells, and particularly morphology of one part of their siliceous cell walls (the valve), that is best known in diatom biology, because these microarchitecturally rich structures have been the basis of species identification for the ca. 10,000 species currently described. The sexual stages, on the other hand, are relatively well known for only a few species, possibly no more than 0.1% of the estimated 100,000 diatom species [3], despite the fact that both sexual and vegetative stages are subject to evolutionary processes.

Sexual reproductive characters (e.g., structures, processes) are strongly conserved across a wide range of biota. As such they are often used to infer deep divergences within a variety of higher level taxa, for example floral structures in flowering plants [4], sexual spores in fungi [5] and reproductive organs in various insect groups [6]. Sexual reproductive structures and processes are known in any detail for only a small number of diatom species, and the entire life history is known in fewer still. So limited understanding of diatom sexuality leaves this potentially fruitful aspect of their evolutionary biology virtually unexplored.

**Fig 1:**
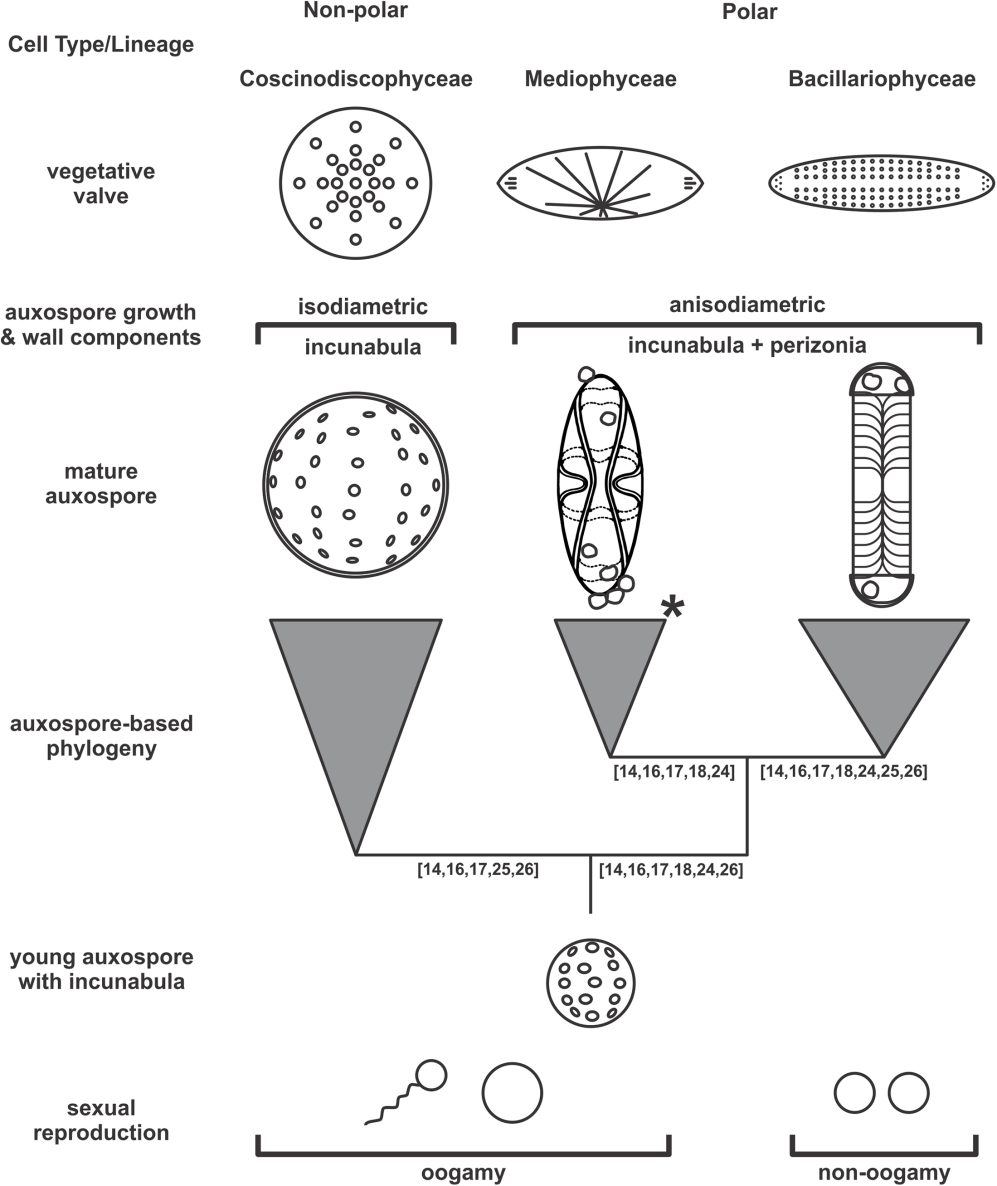
Schematic summary of the deep divergences in diatoms using auxospore type to define each of the branches. Numbers below individual branches indicate references to molecular phylogenies recovering monophyly for these branches [14, 16–18, 24–26]. Auxospores and valves are schematically simplified and not intended to be attributed to any individual species. Asterisk (*) in Mediophyceae indicates exception for non-polar Thalassiosirales who lost perizonia secondarily; see explanation in text. Mediophyceae perizonium reprinted from [9] under a CC BY license, with permission of Koeltz Scientific Books, original copyright 1982.

The auxospore is a cell type unique to diatoms and is integrated into the sexual means of large cell size restitution [2, 7, 8]. It has proven to be evolutionarily informative, providing insights into deep, at times unanticipated relationships among the diatoms [9–12]. Auxospore growth patterns and cell wall structures segregate diatoms into two major groups, consistent with those recovered by SSU-based molecular phylogeny [13–16], cox1 [17], and more recently also in multigene trees [18]. There, diatoms with isodiametrically growing auxospores and incunabula in their walls (one group of centrics) are separated from those growing anisodiametrically with walls containing incunabula and perizonial bands (remaining centrics and pennates; Fig 1). The first group develops circular (or non-polar) vegetative valves, while the vegetative valves of the second group have more complex outlines (elliptical, elongated, quadrangular, etc.) in both centrics and pennates. These complex outlines are facilitated by greater expansion of the auxospore in areas not limited by bands (perizonia), among other factors. The sole currently known exception in the published record to the latter are Thalassiosirales, with circular valve outlines. However, thalassiosiroids are repeatedly shown as a sister-clade to the polar centrics, e.g., Lithodesmiales ([14, 18–23] and others) indicating that the group most likely lost their auxospore wall bands secondarily upon divergence from the last common ancestor with a polar centric diatom. As such, they are an excellent example of the shortcoming of purely-morphology based [21] phylogenies. The auxospore based phylogeny contrasts with the two earlier most commonly used systems that divide diatoms into centrics and pennates (with flagellated and non-flagellated male gametes, respectively) or centrics and two classes of pennates based principally on valve face morphology ([2] and references therein). We support the hypothesis [9, 14] that novel characters in the auxospore wall and development in the ancestors of polar centrics facilitated the departure from the simple circular valves to more complex shapes that eventually culminated in the emergence of bilateral pennates with a sternum.

Members of the *Paralia sulcata* (Ehrenberg) Cleve species-complex are ancient, widely distributed diatoms, readily recognizable in the sedimentary fossil record and in the coastal seas of today. Some species of *Paralia* are known from the Upper Cretaceous (e.g., *P*. *crenulata* [Grun.] Gles., ~84–66 Ma [27–30] until the Lower Oligocene, ~31 Ma [28], while various others persist from the Cenozoic (since ~66 Ma) to recent, attesting to the evolutionary success of the lineage. Depending on the gene sequence and analysis, *Paralia* species join one of the basal clades of the non-polar centric diatoms [14, 18, 31], the Coscinodiscaceae (*sensu* [14]). This genus is currently represented by at least three extant, genetically distinct, morphologically cryptic or semicryptic species complexes, *P*. *sulcata/fenestrata* Sawai & Nagumo, *P*. *marina* (W. Smith) Heiberg and *P*. *guyana* [32]. Similar to most other diatoms, in all these species only the valves representing the vegetative part of the life histories are relatively well known. However, even these are generally limited to smaller cell-size class specimens, rather than inclusive of the morphological variation over the entire range of the species-specific frustule size. To date, sexuality has not been observed in any member of the genus, despite its antiquity. Examining *Paralia* sexual stages may hold promise of informing on the evolutionary significance of sex-related characters in one of the oldest extant diatom genera.

The aim of this paper is twofold. First we describe the process of auxosporulation in the non-polar centric diatom *Paralia guyana* MacGillivary and document the origin of the initial cell. Second, we present the structure of the initial and early post-auxospore cells and discuss them in the context of currently held views on diatom evolution, their fossil record and molecular phylogenies.

## Materials and Methods

### Establishment of monoclonal cultures

Seawater and sediment samples were collected from intertidal sites on the Canadian Atlantic and Pacific coasts of North America. All collection sites were unrestricted public access areas and given that only small volumes (< 2 ml) of seawater or sediments were involved for each sample, no specific permissions were required for these activities, and no endangered or protected species were involved. Single chains of *Paralia* were isolated from these samples to establish monoclonal cultures as described in [33]. All cultures were grown in f/2 growth media [34] at 12–16°C at an irradiance of 40–50 μmol photons m^-2^ s^-1^, 12:12 Dark:Light (D:L) cycle, and inoculated into fresh media approximately every 6 weeks. The three clones examined were: West1C2 (from Aberdeen, WA, USA; 46°58’31.34”N, 123°48’56.59”W), GP1 and GP3 (both from Grove’s Point, Cape Breton, Canada; 46°13’N, 60°20’W).

### Fixation, staining and light microscopy

To visualize nuclei during auxosporulation, cells were fixed with 2.5% (v/v) glutaraldehyde in seawater (final concentration) and stained with DAPI (4’, 6-diamidino-2-phenylindole) according to [35] with minor modifications. Chloroplasts were bleached with 10 ml 99% methanol followed by 5 ml Tris buffer. Following bleaching, 0.1 μl DAPI (10 μg/ml) was added and incubated in darkness at 2–3°C for another 24 hours prior to examination.

Brightfield and DAPI fluorescence light microscopy was performed using two Zeiss microscopes as required (Carl Zeiss, Göttingen, Germany). A Zeiss Axioskop 2 Plus fitted with a cooled AxioCam color camera, HBO 100 fluorescence illuminator and Filter Set 01 was used for reconnaissance work, while a Zeiss AxioImager.Z2 microscope with a cooled AxioCam MRm monochrome camera, Colibri LED fluorescence illuminator (365 nm LED) and Filter Sets 62HE and 49 was used for in-depth investigation. Monochrome fluorescence images presented here were pseudo-colored appropriately based on the filter used for acquisition.

### SEM imaging and energy dispersive X-ray spectroscopy (EDS)

Auxospores and frustules from each of the cultures were subjected to SEM examination on flat polycarbonate filter as in [36] or a grooved LP substrate as in [37], as appropriate. Specimens were examined using a JEOL JSM-5600 SEM (JEOL USA, Peabody, MA, USA) at the Digital Microscopy Facility, Mount Allison University, operating at 10 kV and 8–20 mm working distance. Diameters of cells in auxosporulating cultures were measured using dmfMeasure software [38]. A complete metric data set for the clones is available in [32]. Valve structure terminology followed [33] and [39] while the structures associated with auxosporulation were named following [7].

EDS was performed with the same instrument equipped with an Oxford Inca Energy 200 EDS system and at 20 mm working distance. Since the only element of interest in this study was silicon (Si-K_α_, X-ray energy 1.74 keV), an accelerating voltage of 10 keV provided sufficient overvoltage for efficient X-ray excitation. Spectra were acquired for 100 s (dead time corrected) at 0.1 nA beam current, energy range 0–10 keV into 1024 channels. The EDS spectra were collected from intact and unobstructed structures and/or auxospores. Spectra from the polycarbonate support filter adjacent to the auxospores were also routinely taken and showed no remote excitation from neighboring siliceous components (if present) at distances as close as 3 μm.

## Results

Auxosporulation was examined in three clones of two genodemes after three or four years in culture (depending on the clone). In one, the “*servidei*”-genodeme the process has continued for 28 months (at the time of this writing, September 2015). In the two other clones, the *“capebreton*”-genodeme, auxospores were detectable for about one year. These clones represented only three out of nine genetically identical clones (in markers examined, [32]) that were auxosporulating. Stages of auxospore development observed in LM and SEM were the same, irrespective of the genodeme. Most images presented here come from the West1C2 clone because it was the most prolific in auxospore production.

**Fig 2:**
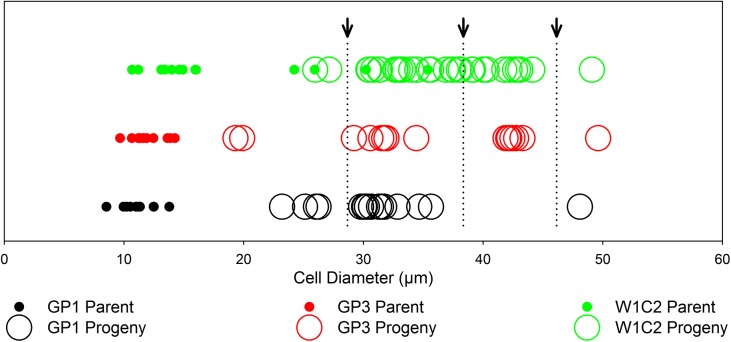
Cell sizes of the *Paralia guyana* parent and progeny individuals in the three auxosporulating clones. Small filled circles indicate individual parental cells, unfilled circles represent progeny cells. Note three or four size-classes of the progeny cells (depending on clone). Top arrows point to the discontinuities between cell size-classes representing progeny cells of the consecutive rounds of auxosporulation. Note that some of the large cells in the clone West1C2 designated as parents (full circles) are vegetative cells derived from initial cells produced by previous round(s) of auxosporulation (by the smaller cell-size parents) and who became parents producing the next generation of still larger auxospores and initial cells.

The “*servidei*”-genodeme clone West1C2 underwent at least three rounds of auxosporulation within about two years of observation, each resulting in a progeny size at least 10 μm larger than that of their parent cells. The first auxospore parent cells were 9–16 μm in diameter while their initial cells were ~20+ μm in diameter. Then, after mitotically dividing for some time, these larger cells underwent their own (the second round) of auxosporulation and produced initial cells of ~35+ μm in diameter. Finally the second set of large cells produced a third set of auxospores and initial cells approximately 10 μm larger than their own parents (the third round of auxosporulation); thus the largest initial cells were ~50 μm in diameter. A similar step-wise process likely occurred in two clones of the “*capebreton*”-genodeme (GP1 and GP3), judging from the larger progeny cell size-class distribution (Fig 2; note three arrowheads at the top pointing to discontinuities in cell-size classes indicating one, two and three cohorts of progeny). Parental (P) and initial cell (IC) metric characters are given in S1 Spreadsheet.

### Observation of intact cells

**Fig 3:**
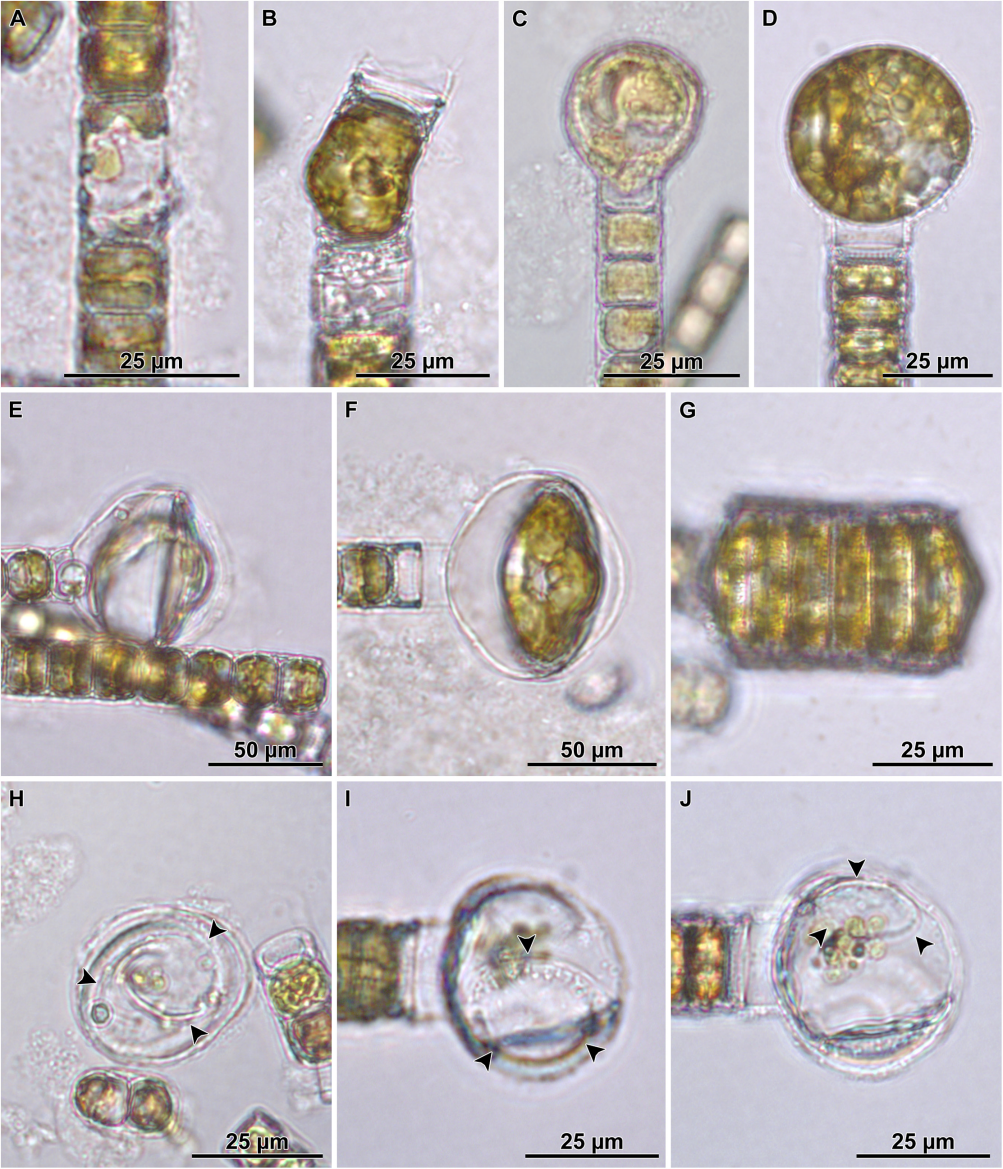
Auxospore enlargement as observed in live material and brightfield microscopy. A—elongated cell represents auxospore development stage before it rounds up; B—auxospore with initially uneven lateral expansion; C—a small, almost spherical auxospore; D—near full size spherical auxospore filled up with chloroplasts; E—a nearly mature auxospore undergoing second partial plasmolysis, note small refractive structure present in parental theca, behind retracting initial cell protoplast; F—a mature auxospore with initial cell inside; G—a short filament of post auxospore cells with initial epi- and hypothecae incorporated into the viable end cells; H—expired auxospore, nearly clear of cell contents, showing an outline of initial epitheca (arrowheads); I-J—another deteriorating auxospore, nearly clear of cell contents at two different foci: I—focus on an initial epivalve, (arrowheads); J—focus on a large scale (arrowheads).

When observed in brightfield preparations, auxospore development began with the elongation of the sexualized cell (Fig 3A and 3B). This was accomplished by deposition of a greater number of cingulae to form a long cylindrical cell that exceeded the pervalvar length of typical mitotically dividing cells. Then the protoplast took up a spherical form, and swelled out from the confines of the parental frustule (Fig 3C and 3D). It became clear that the auxospore at this stage was surrounded by a transparent (likely richly organic), thick cell wall. A portion of the wall and the protoplast often remained inserted into one, and sometimes both, parental thecae, resulting in imperfectly spherical cell outlines in the earlier stages of expansion. Chloroplasts could be seen filling up the peripheral area of the auxospore during the expansion, even in very large cells (Fig 3D).

Fully expanded auxospores were spherical. Then, the auxospore protoplast underwent a first partial plasmolysis and the initial epivalve developed (Fig 3E). This was followed by a second plasmolysis, at the opposite pole of the auxospore and the deposition of the initial hypovalve (Fig 3F) and divisions of the initial cell followed (Fig 3G). The plane of initial valve deposition varied somewhat (Fig 3E,3F and 3H–3J), but it was most often in a great circle (orthodrome), resulting in circular valves. In other cases the valves were deposited at an oblique angle to the great circle, resulting in slightly elliptical valves. This was most often observed in the “*servidei*”-genodeme clone West1C2. The slightly elliptical variant in initial valve outline appeared to be corrected back to circular outlines quickly thereafter. We looked for, but did not find chains with consistently elliptical frustules, such as those documented by e.g., [30].

**Fig 4:**
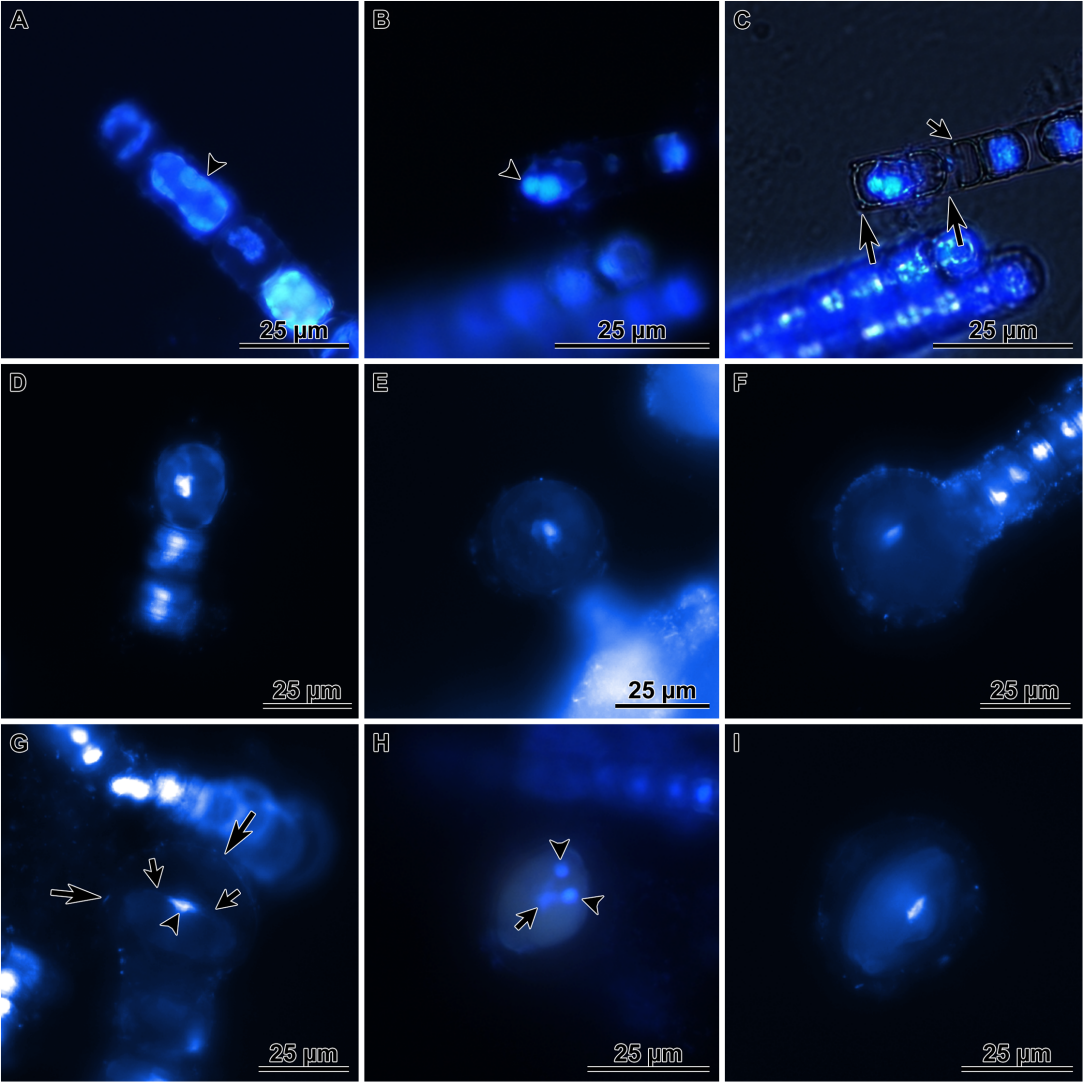
Auxospore nuclei as observed in DAPI stained cells. A—elongated cell stage of auxospore development, note cell nucleus divided and the smaller of the sister nuclei is undergoing pyknosis (arrowhead); B & C—the same cells shown in different illumination, another elongated cell with one functional and one pyknotic nucleus (two large arrows point to the domed valves of this cell), note a smaller cell below the young auxospore in C (small arrow points to the flat hypovalve of a smaller frustule (see also SEM figures below); D—nearly spherical, small auxospore with centrally located nucleus; E—larger, spherical auxospore with centrally located nucleus; F—nearly full size auxospore with centrally located nucleus; G—nearly mature auxospore undergoing first partial plasmolysis prior to depositing the initial epivalve, note position of the nucleus (arrowhead) immediately underneath the retracting protoplast surface (small arrows) and a dome-shaped auxospore wall above (large arrows); H—mature auxospore after second partial plasmolysis, note two compact pyknotic nuclei located peripherally (arrowheads) and one functional nucleus located deep in the cell (arrow); I—mature auxospore with initial frustule, note nucleus near cell center.

DAPI stained cells informed on the nuclear processes during auxospore development. We searched for, but did not find evidence of meiosis in any cell examined. We also did not find evidence of spermatogenesis or flagellated sperm. In the earliest, elongated-cell stages of auxospore development, the DAPI stained nuclei were swollen and then divided. A binucleated cell resulted from this division, but shortly thereafter (before the sibling nuclei pulled far apart) one of the nuclei pyknotised (Fig 4A–4C). These pyknotic nuclei presumably degraded quickly because all expanding (spherical-cell stage) auxospores and plasmolizing cells observed carried only one nucleus (Fig 4D–4G). In only a few cases, we observed that preceding production of the cell destined to become the auxospore, an auxospore mother cell (AMC) divided unevenly (rather than turning directly into an auxospore), producing a cell that would become the auxospore and a small, anucleate residual body (Fig 4C). This division produced one normal hypovalve encasing the binucleated cell that became an auxospore, while the residual body was enclosed by one of the AMC theca and by a rudimentary, lightly siliceous hypovalve.

Multinucleated cells could be seen again in full size auxospores when initial valves were being laid down. We presume that each initial valve formation was preceded by successive acytokinetic mitoses, each associated with pyknosis of the supernumerary nucleus. A trinucleated cell was recovered when two pyknotic nuclei were peripherally located while the large (presumably functional) nucleus held a central position between two initial thecae (Fig 4H). The uninucleate initial cell (Fig 4I) divided soon after the initial frustule was completed (Fig 3G). No resting period for initial cells was observed.

Some LM images suggested the existence of at least one large scale in auxospore walls deposited during the time of their enlargement (Fig 3J). The clearest documentation of such structures comes from the LM imaged auxospores that did not complete their development and lost their protoplast (Fig 3H–3J), so that these structures became notable. SEM examination documented one such scale as well (see below), but it was impossible to determine in that case whether the auxospore was viable. Consequently, it is difficult to determine at this time whether large scale formation is a normal step in the course of auxospore development, or whether they forecast abnormal development in moribund cells.

### Observations of cleaned cell walls

**Fig 5:**
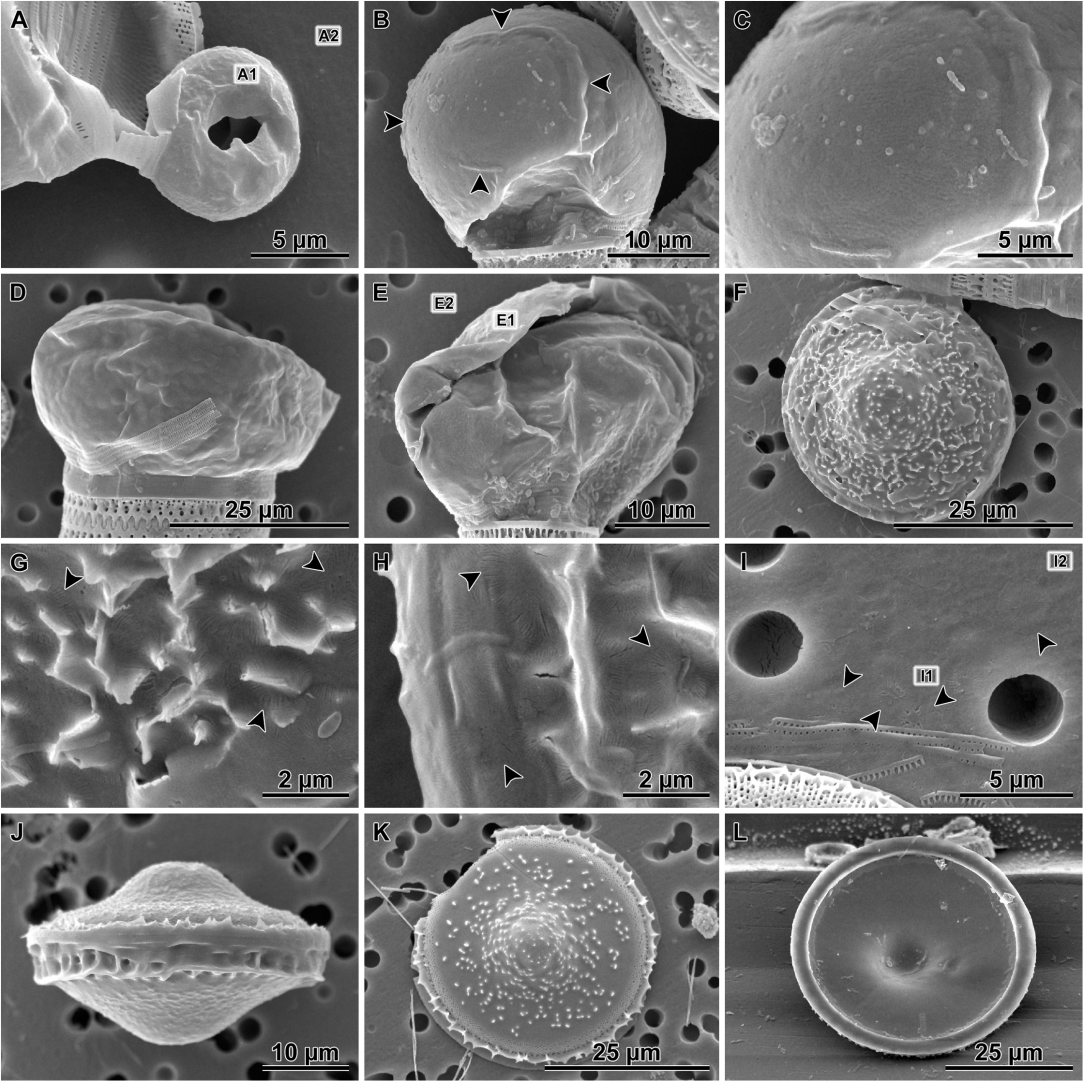
Auxospores and initial cells as observed in SEM; A-I water rinsed, and J-L acid-cleaned specimens. A—theca resulting from uneven division of the auxospore mother cell (compare to Fig 4C), note doughnut-shaped, lightly silicified rudimentary hypovalve (valve EDS spectrum shown in Fig 6A-A1-2); B—a young spherical auxospore with large scale (arrowheads) exposed (compare to Fig 3I and 3J); C—the same large scale showing detail of pitted ornamentation; D—partially collapsed, large auxospore probably captured during partial plasmolysis, note large diameter of parental theca demonstrating the second round of auxosporulation; E—a partially damaged auxospore with thick wall with siliceous elements (EDS spectrum shown in Fig 6B-E1-2), note that the small parent cell of this auxospore indicates first round of auxosporulation; F- spinose initial valve covered with thin and pliable remains of the auxospore wall; G—the same specimen as in F at greater magnification showing scaly incunabulae (arrowheads) in the auxospore wall; H—another initial frustule enshrouded in the auxospore wall demonstrating scaly incunabulae in the girdle region (arrowheads); I—individual incunabular scales (arrowheads) disassociated from the auxospore wall (EDS spectrum shown in Fig 6C-I1-2); J—complete initial frustule in girdle view, note epicingulum; K—external view of an initial valve; L—internal view of an initial valve.

Small heterovalvar frustules were also seen in SEM preparations (Fig 5A), albeit not as frequently as in LM (Fig 4C). Auxospores (Fig 5B–5I) and large heterovalvar frustules (initial cells, Fig 5J–5L), on the other hand were relatively common. Auxospore walls were relatively thick (Fig 5B,5D and 5E) and throughout much of the cell expansion stage occurred outside the parental theca. The thick walls likely contained much organic matter because even mild and short duration acid-based cleaning for SEM proved destructive to their integrity. Thus all the auxospores shown here with fairly intact walls came from preparations where cells were only rinsed with distilled water. This protocol resulted in a somewhat lower quality images compared to those obtained from acid cleaned samples typically employed in studying diatoms.

**Fig 6:**
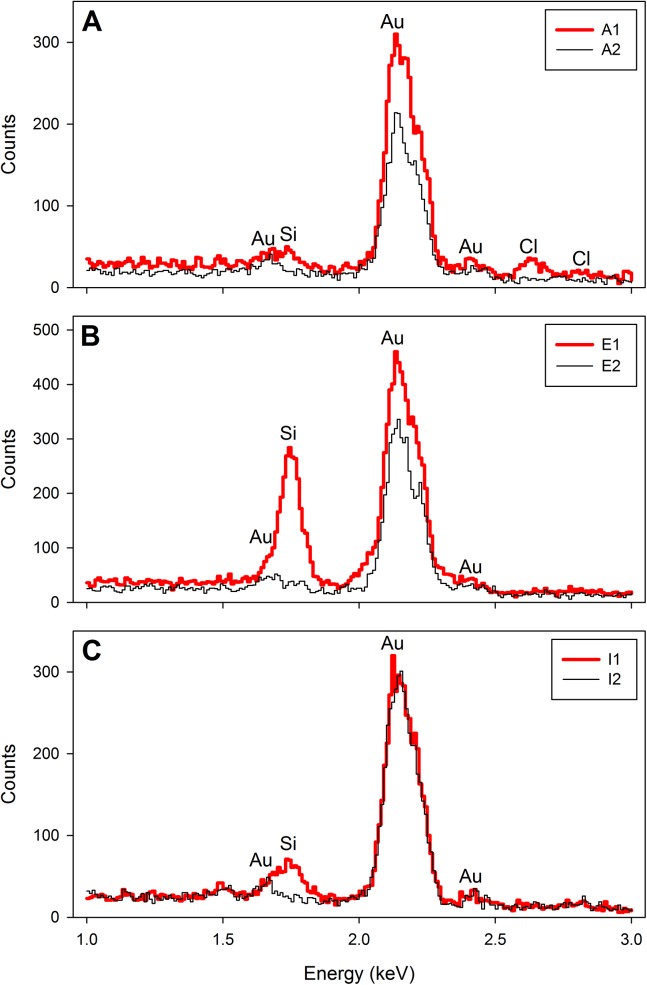
Representative EDS spectra. Spectra acquired from locations shown in Fig 5A, 5E and 5I, respectively for vestigial hypovalve, auxospore wall and incunabular scale (locations indicated by boxes). Note difference in vertical scale of the spectra in Fig 6B. Spectra from position 1 are taken from structure shown in Fig 5 and spectra from position 2 from filter substrate which shows no detectable silicon peak. Gold peaks were generated from the conductive coating.

Vestigial hypovalves were found in cells resulting from uneven division of the auxospore mother cell. They were doughnut-shaped and very lightly silicified (Figs 5A and 6A-A1-2), relative to the walls of expanding auxospores (Figs 5E and 6B-E1-2) or an individual incunabular scale (Figs 5I and 6C-I1-2). The shape integrity of the walls of young auxospores was also facilitated, at least in part by numerous small, siliceous, rounded incunabular scales (= 2.1 μm, other statistics in S1 Spreadsheet; Figs 5G–5I and 6C-I1-2). The surface of these scales was marked with dichotomizing ribs radiating from a structureless central area (Fig 5I). Some scales had 1–3 small, centrally located pores. In addition, a large, gently domed scale (14.4 x 19.8 μm in short and long axes, respectively) with more complex patterning was seen in one auxospore imaged using SEM (Fig 5B and 5C). Similarly sized structures were more frequently observed in LM preparations (Fig 3I and 3J). This large scale carried stria-like parallel rows of shallow, pore-like depressions. Some or most of the incunabular scales were likely shed off the wall due to stretching of the peripheral organic wall-layers as the auxospore grew in diameter, because only a few layers of small incunabular scales were found associated with mature auxospore walls (those containing initial cells). In mature auxospores, the organic wall component holding scales together was pliable and thin (Fig 5F–5H). When shrouding the initial valve surface endowed with prickles and ridges, the auxospore walls (including siliceous scales) were perforated by these surficial microstructures.

**Fig 7:**
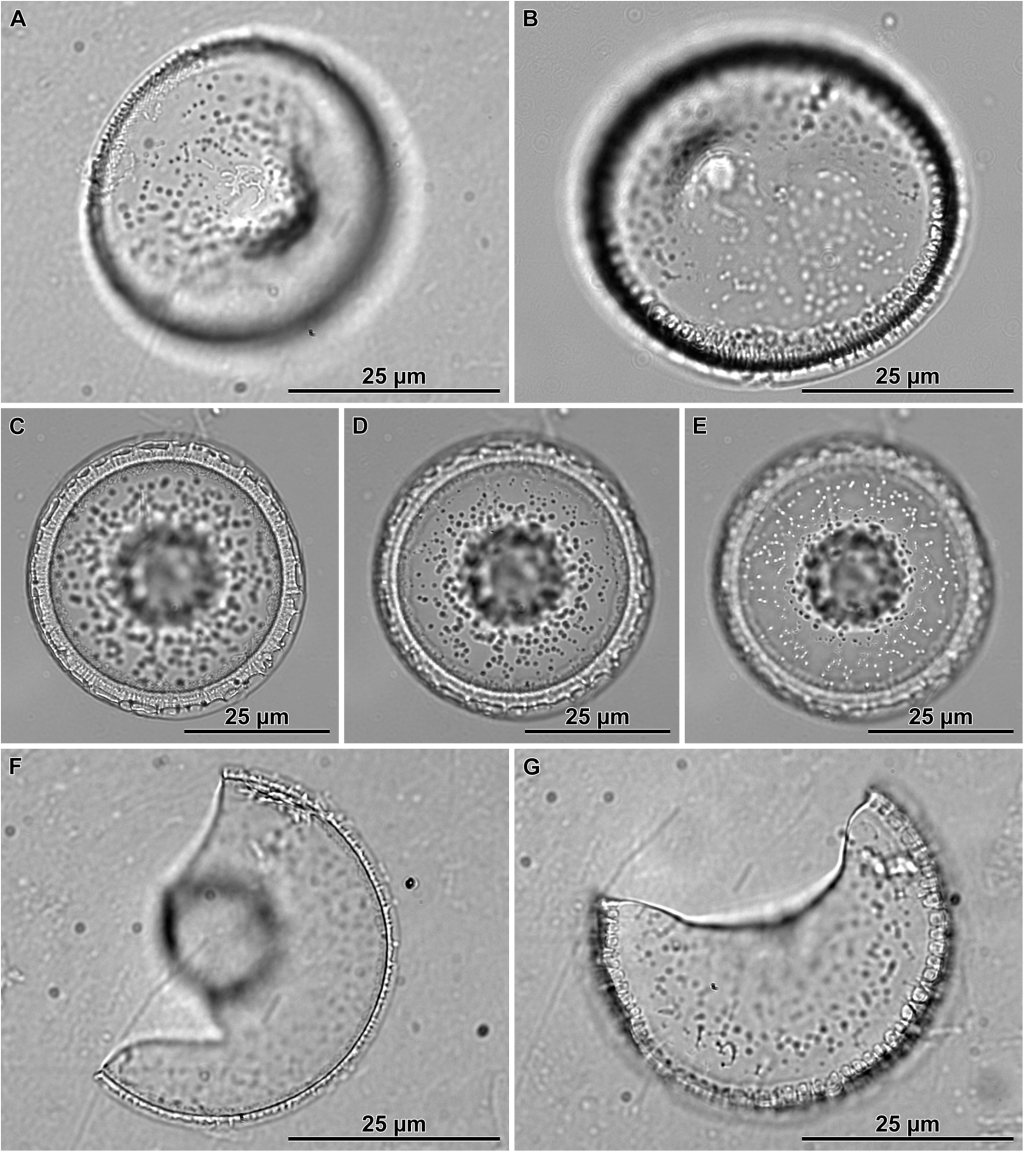
Acid cleaned initial valves observed in brightfield LM. A & B—convex, relatively plain initial valves with single (A) and double (B) elevations, note band of quincunx pores at the valve margin; C through E—a strongly concentrically undulated valve at three different foci: C—focus on valve margin and striae; D—focus on peripheral part of the valve face, ribs and stout spines; E—focus near central elevation with smaller spines; F—inside view of initial valve with central elevation; G—outside view of a less convex initial valve, note ribbed margin.

**Fig 8:**
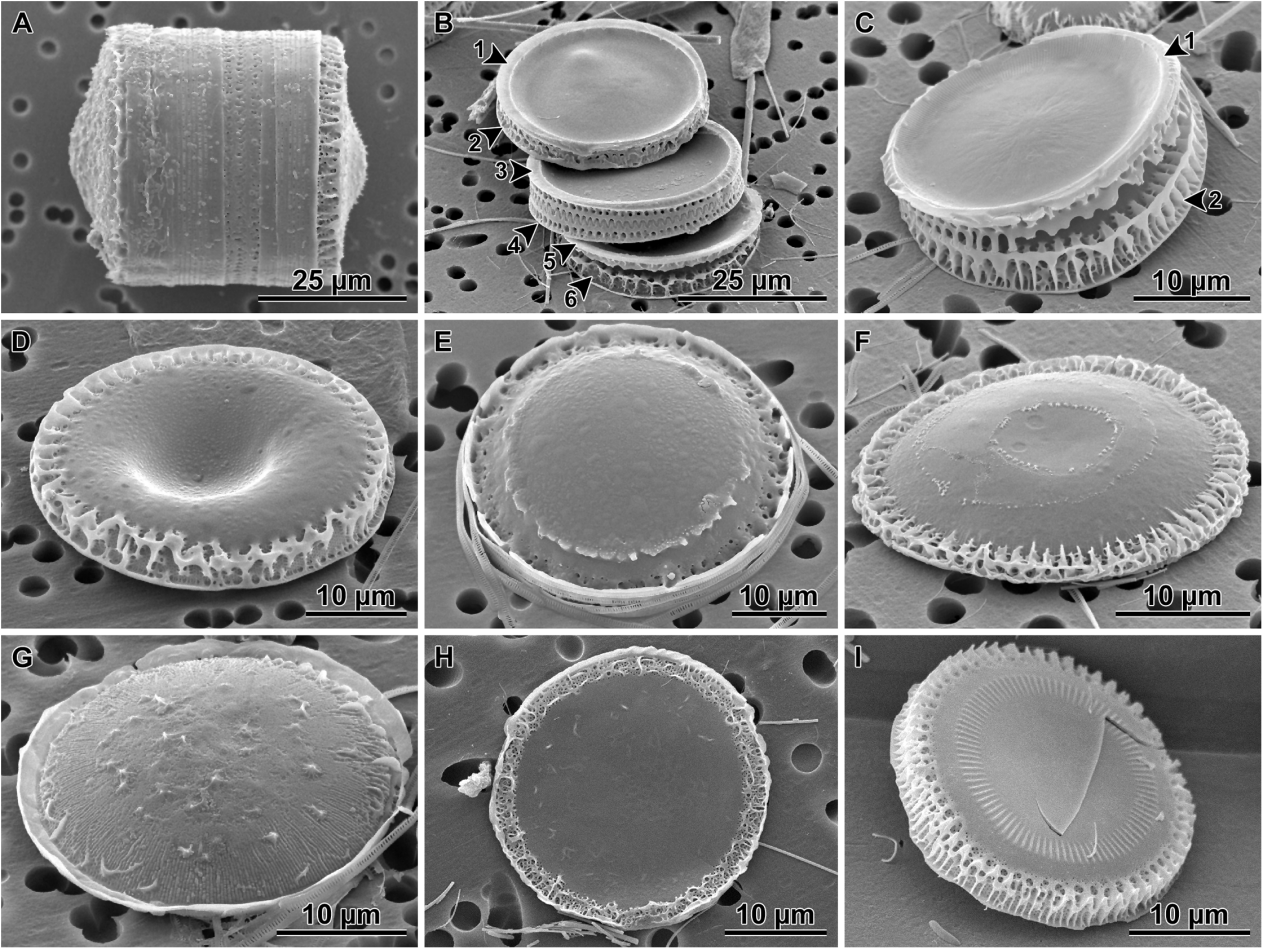
Post auxospore frustules and valves as observed in SEM. Morphology is intermediate between initial and typical vegetative valves. A—a short chain (probably 3–4 cells), note strong cingulae and initial valves on the end cells; B—disarticulated post auxospore chain showing 6 valves with imperfect marginal linking spines and mantles, note varying degree of imperfection likely contributing to weakness of cell-linking, thus the need for strong cingulae; C—two post-auxospore valves with mismatching marginal linking spines; a sibling valve at top (arrowheads) also with atypically shallow mantle and malformed linking spines; D—external view of a post auxospore valve with concentric depression and underdeveloped, imperfectly ornamented mantle; E—domed post-auxospore valve with imperfect internal and external linking spines; F—relatively flat post-auxospore valve with concentric depression, shallow mantle and malformed linking spines; G—post-auxospore valve with pronounced radial striation, featureless flange and spiny protrusions; H—somewhat elliptical post-auxospore valve with network of peripheral thickenings; I—typical intercalary vegetative valve with incompletely silicified mantle, note typically narrow fenestra which in more strongly silicified valves are covered by siliceous outcroppings, obscuring fenestra in SEM images.

The initial cell frustule was strongly silicified and heterovalvate (Fig 5J) with valves circular in most cases (Fig 5K and 5L). The initial and post-auxospore valve architecture differed from that of typical vegetative valves in a number of characters in both external and internal structures (Figs 7 and 8). Initial frustules were notably asymmetric in valvar plane, with one valve face more gently convex while the other was concentrically undulated bearing 1–2 steeper central elevations (Figs 7A–7G and 8A). Each initial valve face contained a peripheral, flatter flange and then a mantle (Fig 5K). Up to seven copulae (Fig 5J) were observed in initial epithecae. Valve copulae were narrow and perforated by short, parallel slits, 31–42 slits in 10 μm in copulae associated with initial and vegetative cell valves (see S1 Spreadsheet for other statistics).

Both valves of the initial cell were sculptured and ornamented. They carried spines, prickles, ridges and flap-like protrusions throughout and varied in size and density among individuals of the same clone (Figs 5F,5J,5K, 7A–7G and 8A); full morphometric analysis of the parental clones is also given in [32]. A band of pore-areolae occupied a narrow area of the valve face and the mantle (Figs 5K and 7A–7F). Pore-areolae were organized in a quincunx pattern; 27–44 in 10 μm (other statistics in S1 Spreadsheet). Pores formed radial rows, traceable further up the valve face than pores themselves were apparent. Rimoportulae were absent on at least one and possibly both initial valves.

**Fig 9:**
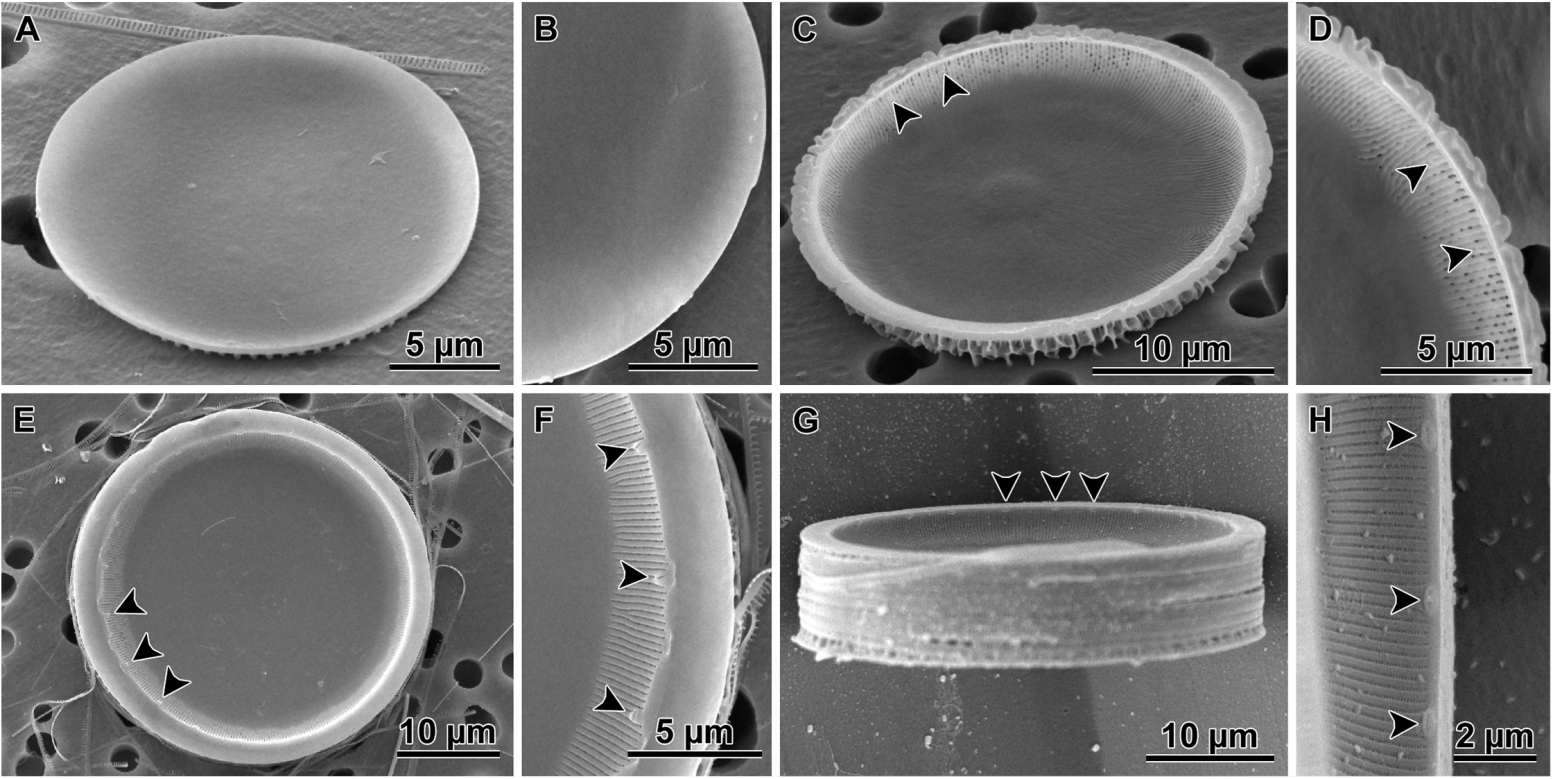
Internal view of initial and post-auxospore valves as observed in SEM. A & B—note absence of rimoportula; C & D—rimoportulae near the valve mantle edge aligned perpendicularly to the mantle rim (arrowheads); E & F—hammock-shaped rimoportulae (arrowheads) with internal openings variably oriented with respect to the mantle rim; G & H—low-profile rimoportulae in typical position for a vegetative valve (arrowheads), underneath the mantle rim and parallel to rim’s circumference (focus is on the distal rim). Note imperfect striation and external ornamentation of the mantle on some of the valves.

**Fig 10:**
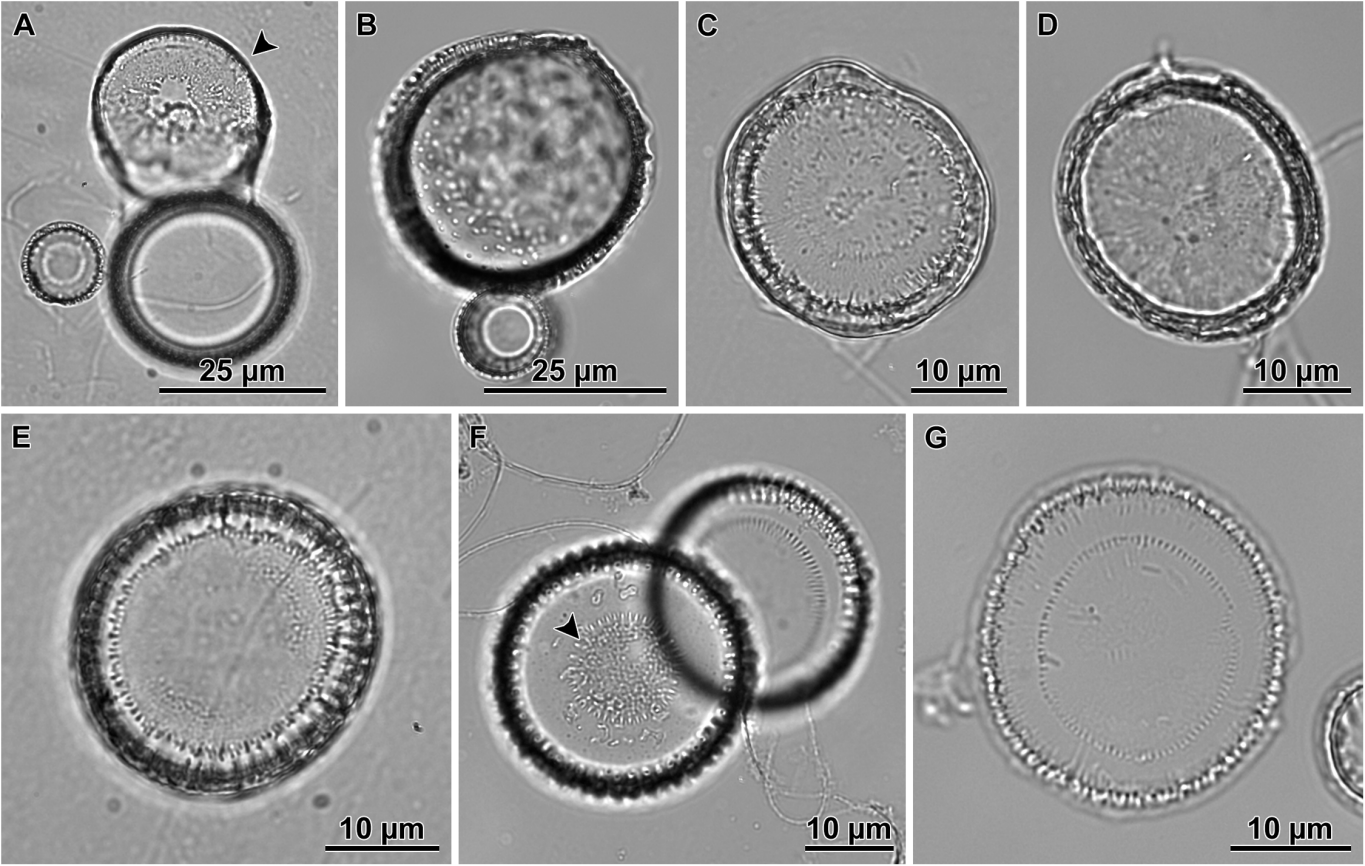
Acid cleaned post auxospore valves in brightfield LM. Morphology is intermediate between initial and typical vegetative valves. A—valve face with irregular rugose markings and narrow, striated rim (arrowhead); B—valve face with numerous spines dispersed about the valve face; C—face with irregular marginal and central radiate shallow ribs, not spines; D—slightly elliptical valve face without internal linking spines and irregularly thickened margin; E—slightly irregular valve with ill-formed marginal spines and puncta; F—valve with well-developed marginal region, but with irregular central ornamentation (arrowhead); valve below is a typical vegetative intercalary valve; G—valve with relatively well developed internal linking spines but underdeveloped marginal spines.

Initial cells divided producing more heterovalvate frustules (Figs 3G and 8A). Filaments up to 32 cells long were observed, capped with live heterovalvate end-cells where each possessed one of the initial valves. This demonstrated the degree of persistence of the heterovalvate end-cells with initial thecae. Valves with a range of morphologies intermediate between the initial valves and typical vegetative valves were found (Figs 8–10) and they included: valves with faces carrying depressions and elevations (although less pronounced than on the initial cell valves; Fig 8D–8F), with malformed marginal linking spines and imperfect fenestra (Figs 8A–8H and 10A–10G), flat (or nearly flat) valve faces (Fig 8G and 8H) without or with imperfect internal linking spines (Figs 8D–8I and 10A–10G). Building a typical vegetative valve in our diatom also required forming, re-positioning and re-orientation of the rimoportulae (Fig 9A and 9B). Imperfectly constructed post-auxospore valves were recovered with the rimoportulae external slit orientation varying with respect to the valve mantle distal margin and with stalked, hammock-shaped rimoportulae that were not located underneath the mantle rim overhang (Fig 9C–9F). This contrasts with sessile, low profile rimoportulae tucked under the valve mantle rim that are parallel to the mantle rim and perpendicular to the striae in typical intercalary valves (Fig 9G and 9H).

Mantle fenestra, outcroppings and arches supporting marginal linking spines form a superstructure overgrowing the basal silica layer in the mantle region of the normal vegetative valve and the valve face periphery, often obscuring the quincunx pore pattern on the basal silica layer (Fig 8I). Typical intercalary and separation valves were also found, with their valve faces being smooth to strongly prickly, with or without internal linking spines, as expected in respective genodemes. The typical vegetative valve structure, therefore, developed gradually, over several mitotic divisions and produced intermediate forms and heterovalvate frustules which were progressively less resting spore-like but increasingly similar to a typical vegetative frustule.

## Discussion

### Comparison to other taxa

Auxospore production in diatoms may involve a variety of processes [7]. Here, based on the absence of evidence of meiosis, and spermatogenesis in particular, we doubt that auxosporulation observed in *P*. *guyana* is oogamous, as would be expected in a allogamously reproducing centric species. We postulate that the process observed in *P*. *guyana* most likely represents apomixis [7]. Three facts support this conclusion. First, the cells resulting from enlargement were clearly initial cells produced inside auxospores with scaly envelopes [7] typical of non-polar centrics, in contrast to vegetative cell enlargement [7, 35, 40] where no auxospore is produced. Second, only one nuclear division in the earliest, elongated cell-stage of auxospore development was consistently observed and it was acytokinetic with pyknosis of one of the division products. Third, only one nucleus was found in all expanding auxospores observed, calling into question the existence of a second meiotic division. Apomixis is the simplest of the processes explaining our observations, similar to that reported for other centrics ([8] p. 262; [9] p. 129) and pennate diatoms [8, 41]. A less likely possibility, the extremely reduced form of autogamy [42], could not be completely discounted, because the second nuclear division (meiosis II) and subsequent fusion of haploid, sister nuclei might have been very rapid and thus the trinucleated stage (two sister nuclei following second meiosis plus a lasting pyknotic [homolog] product of the first division) in the young (small) auxospores difficult to capture. However, given the considerable number of auxospores we examined, reduced autogamy seems less likely.

Auxospore expansion stages and wall composition in *P*. *guyana* were similar to other non-polar centric diatoms [7, 8, 10, 14, 43–48], which are the same irrespective of the underlying sexual processes (allogamy, autogamy or apogamy). The final stage, the development of the heavily silicified initial cell, was relatively lengthy compared to such diatoms as *Thalassiosira angulata* (Gregory) Hasle or *Tabularia fasciculata* (C. Agardh) D.M. Williams & Round ([42, 49] respectively), and took days rather than hours. This lengthy final stage allowed numerous older auxospores to accumulate in the culture, rendering this stage suitable for detailed SEM examination. Neither the singular nor the “multi-step” auxosporulation observed in our cultures resulted in restoration of the reported species-specific maximum cell size progeny of ca. ~80 μm in diameter ([50] p. 276; [51] p. 34).

Auxosporulation in Paraliales Crawford [2] is poorly known compared to related non-polar centrics (e.g., *Melosirales* [43–46, 50]; *Stephanopyxis* [8, 47, 48]), some of which might have practiced alternation of vegetative cell-size diminution and auxosporulation since the Upper Cretaceous (~ 70–73 Ma; [52]). Among Paraliales, auxospore development and structure has only been examined in *Ellerbecki*a *arenaria* (Moore ex Ralfs) R.M. Crawford [53], the other genus in the order [2]. There are notable similarities between the auxospores of these two taxa, but there are also many features in structure and development of the initial cell in *Paralia* that are not known in *Ellerbeckia* or any other diatom examined to date.

Similar to auxosporulation in *E*. *arenaria*, we also found residual, nucleus-free cytoplasm remnants associated with the earliest stage of auxospore development, albeit this was observed only sporadically in *P*. *guyana*, and not in all auxospores as in *E*. *arenaria*. A doughnut-shaped, siliceous structure similar to the “oogonial wall” (*sensu* [53]) was also encountered, but only sporadically in our diatoms, and they were not associated with the auxospore itself, as in *E*. *arenaria*. In *P*. *guyana*, these structures may be best understood as vestigial hypovalves resulting from an uneven mitotic division of some auxospore mother cells (AMC). In these cases, the cell that was to become the auxospore received nearly all of the protoplast (including a pyknotising supernumerary nucleus) and deposited a fully silicified hypovalve before beginning isodiametric expansion. The anucleate product of the uneven cell division (residual body) retained one of the AMC valves and deposited a doughnut-shaped, weakly silicified vestigial hypovalve (compare EDS spectra peaks for Si in Fig 5A to Fig 5I). However, many auxospores developed directly from the elongated cell without uneven cytokinesis or rudimentary-valve formation because we only infrequently observed residual bodies and vestigial hypovalves associated with the auxospores when they remained attached to their native filaments. Thin, rudimentary valves produced during gametogenesis are known in some other centrics, though only during spermatogenesis [7, 8].

The auxospore wall in both *P*. *guyana* and *E*. *arenaria* consists of an amorphous, presumably organic matrix and embedded siliceous incunabular scales, similar to all examined non-polar centrics and the early stages of auxospore walls in polar diatoms [2, 7, 14, 43, 44], including some raphid pennate diatoms [7]. The organic component holds the scales in place and together they are thought to provide both physical strength for protection and elasticity for expansion. In *P*. *guyana* the walls of our older auxospores appeared thinner and less stiff (relative to the smaller, younger cells), as if the former had shed or stretched out some of the wall components during cell expansion. Incunabular scale size, shape and ornamentation in *P*. *guyana* were similar to those seen in other diatoms examined with the use of electron microscopy (*Melosira*, *Aulacoseira*, *Orthoseira* [43, 44], *Coscinodiscus* [54], and others [7]) in all but one respect. At least one large scale (per auxospore) was seen in some preparations when cell orientation and content allowed (LM) or the outermost layer of the auxospore covering was lost (SEM) in our diatom.

### Initial and post-auxospore cell structure and development

In most diatoms examined, the initial valves differ from that of typical vegetative valves in some aspect of their micro-architecture. For example, in *Stephanopyxis* and *Melosira*, relatives of *Paralia* [18], the colony linking spines develop only on valves produced following the first mitotic division of the initial cell [43, 44, 48]. In *Aulacoseira* spp. and *E*. *arenaria*, initial valves also show simplified areolation and are hemispherical rather than cylindrical (as are vegetative valves), among other characters [43, 44, 53]. These structural differences result in heterovalvate cells once the first mitotic division brings about more typical vegetative valves; one valve is an initial while the other carries morphological characters of a typical vegetative valve.

The degree of dissimilarity between vegetative and initial cell valve morphology seen in *P*. *guyana* is exceptional, but not unknown among diatoms. Species of one other non-polar centric diatom genus, *Leptocylindrus*, are known to produce initial frustules radically different from their vegetative counterparts. They clearly result from a sexual process [31, 55], but were thought to function as resting spores [56] in the life history of the species. Indeed, the initial cell frustules of some *Leptocylindrus* species demonstrate structures similar to those seen among resting spores of other diatoms, such as extant *Chaetoceros* species. It is possible that this similarity and the capacity to rest [56] have retarded a full understanding of what they may represent.

Initial frustules of *P*. *guyana* clearly have structural affinities to diatom resting spores. Diatom resting spores also have heterovalvate frustules and a similar type of external valve face microarchitecture (spines, prickles and ridged relief) that may be as poorly organized as they are on the initial valve face of *P*. *guyana*. The most apparent morphological difference between modern resting spores of, for example *Melosira*, *Ditylum* or *Chaetoceros* and *P*. *guyana* initial frustules is their origin within the auxospore instead of the vegetative spore mother cell. The resting spore-like initial cells are larger than their auxospore mother cells in contrast to a resting spore that originates within the vegetative mother cell-walls and thus are smaller than their parents. A vegetative initial cell may produce resting spores, for example, in some species of *Chaetoceros* [55, 57], and these are also smaller than their spore mother cells. Furthermore, initial cells in *P*. *guyana* do not rest but resume mitotic divisions as soon as they are completed, albeit both growth and divisions of the initial cell are slow due to the massive amount of silica needed for such large and heavily silicified frustules, similar to another heavily silicified non-polar diatom [58]. Finally, unlike diatom resting spores that tend to shed their frustules upon excystment [59], the initial cells in *P*. *guyana* incorporate the spore-like initial cell valves into the frustules of their mitotic progeny for up to at least five rounds of mitosis required to produce the chain of 32 cells observed, if all cells divided in synchrony. This is close to the life-span of a normal, individual vegetative valve, estimated to last only through 6–8 divisions [60, 61].

Developing a typical vegetative valve in more derived diatoms thus far examined is comparatively prompt and involves only 1–2 post-auxospore mitoses [42, 62–64]. Building the typical vegetative valve in *P*. *guyana* after auxosporulation is a longer process because it involves several rounds of mitoses resulting in valves with intermediate, atypical morphologies: undulated valves, imperfect internal linking spines, imperfect external linking spines, poorly developed mantle, etc. Furthermore, the building and re-positioning of rimoportulae that took place during the multiple mitoses leading to a typical vegetative valve in *P*. *guyana* is thus far unprecedented among diatoms. The imperfect rimoportulae height and position relative to the valve mantle margin were somewhat similar to the porelliportulae of some extant *Ellerbeckia* species [65, 66] and extinct *Truania* [67]. The position of rimoportulae on post auxospore valves may reflect their ancestral position in the *Paralia-*lineage before they became located in their current position, by the Upper Cretaceous. Rimoportulae in close relatives of *Paralia* (e.g., *Ellerbeckia*, *Hyalodiscus*, *Stephanopyxis*, *Melosira*) are located away from and are not parallel to the valve mantle rim. The degree and persistence of disparity between the morphology of typical vegetative and post-auxospore frustules is previously unknown among diatoms and raises taxonomic and evolutionary considerations.

### Taxonomic considerations

Post-auxospore heterovalvate frustules have been well documented in diatoms, resulting in the initial cells occasionally being described as separate taxa [68, 69]. In general however, significant structural differences between initial and vegetative frustules are infrequent, rarely affecting identification to the genus level, and affect only a few post-auxospore valves. This is not the case in *Paralia guyana*; despite auxospore development and structure that conforms well to that of other non-polar centrics, it produces remarkably unusual initial and post-sexual cells. Had the initial cells been first found outside auxospore walls, it is unlikely that they all would have been thought to belong to the same species as vegetative valves of *P*. *guyana*, a situation similar to algae with heteromorphic stages in the life cycle of a single species.

Even though typical vegetative valves of *Paralia* and *Ellerbeckia* are somewhat similar (discussed in [65, 70] and not repeated here; but see [66]); their initial cell valves are not. In fact, some characters seen in initial valves of *P*. *guyana* suggest a closer relationship of *Paralia* to several other, non-*Ellerbeckia* diatoms. Most notable is a belt of quincunx pore areolae on the mantle and valve face. Similarly organized, simple pore areolae are also found in *Hyalodiscus* [71–73], *Hyalodiscopsis* [74], *Truania* [67] and *Podosira* [2]. Furthermore, *Paralia guyana* initial valves bear clear similarities to valves of *Pseudopodosira*, which have better defined, concentric elevations on the valve face but have a characteristic, similar marginal band of quincunx poroid areolae on the valve face and the mantle [28]. Most of these genera are known since the Upper Cretaceous (*Paralia*, *Hyalodiscus*, *Pseudopodosira*, *Truania*; [28–30, 67, 72]) or the Paleogene (*Podosira*, *Ellerbeckia*; [28, 72]).

Superposed on the basal silica layer of the initial valves of *P*. *guyana* are spines and ridges; all reminiscent of a number of extinct morphospecies thought to represent diatom resting spores, unfortunately known mostly from LM images only. These include species such as Upper Cretaceous *Acanthodiscus antarcticus* Hajos, *A*. *convexus* Hajos, *Horodiscus rugosus* Hajos & Stradner, *Poretzkia* sp. [75], Upper Eocene *Acanthodiscus rugosus* Pantocsek, *Podosira* sp. [76] and Pleistocene *Pseudopodosira kosugi* Tanimura et Sato [77] and others. All share with our diatom’s initial and post-initial valves a circular valve outline, strong degree of silicification, a modest degree of valve perforation, heterovalvy (when known) and/or propensity for surficial spines, flaps and ridges at the valve face margin and/or scattered around the valve face. Similarly, paleo-morphospecies with circular valve outline from the genera *Liradiscus* and *Xanthopyxis* ([78] p. 106, fig 28; and p. 196, Plate 54 figs 1–5, respectively) carry one or the other of the structures seen in *P*. *guyana* initial valves, all absent in *P*. *guyana* vegetative valves. There are even Lower Cretaceous morphospecies with similar ridged valve-morphology (e.g., *Crossophialus* Harwood and Gersonde) which have circular and heterovalvate frustules ([79]; compare plate 3 Fig 3 to Fig 7H). Neither the type nor positions of the rimoportulae (if present) are known for most of these extinct species. The initial frustules of the earliest, extinct members of the genus *Paralia* from the sedimentary record may have been even more similar to resting spores and thus unrecognized for their true identity and the role in the life cycle of *Paralia*, but attributed to genera such as *Acanthodiscus*, *Horodiscus* or *Poretzkia*. Whether any of these taxa represent initial valves of another species currently known only from their vegetative valves requires further study, but structural similarity of all these valves to initial and post-auxospore valves of *Paralia* speaks for the need for such investigations in possibly less vigorously cleaned and non-sieved sediment samples.

The dissimilarity between the vegetative and initial cell valves is even more extreme for the initial frustules of the extant members of the genus *Leptocylindrus* and might have been just as striking among extinct species; e.g., compare the initial cell of *L*. *minimus* Gran to the extinct genus *Pterotheca* [80, 81]. Indeed, if spore-like morphologies were more common among archaic diatoms, some of the Mesozoic silicified “spores” might in fact represent diatoms and/or their initial frustules.

The three "step-wise" auxosporulations observed in *P*. *guyana* resulted in several incremental size-classes of initial and post-auxospore cells, all significantly smaller than the species specific maximum cell-size. The wide range of spore-like initial cell sizes renders them superficially even more similar to resting spores when found independent of auxospore walls and less similar to the initial cells in modern diatoms which generally tend to be close to the specific maximum size for the species. We therefore postulate that in terms of the structure of its initial and post-auxospore valves, *P*. *guyana* expresses some of the characters present in its ancient ancestors and absent in their modern vegetative frustules.

### Evolutionary considerations

Well preserved fossilized remains of Jurassic and Lower Cretaceous diatoms are rare. The Jurassic *Pyxidicula* spp. (Liassic ca. 190 Ma; [82, 83]) are generally thought to represent the oldest diatoms, although not without reservations due to their non-conformance to the design of modern diatom vegetative frustules (comprised of nearly identical thecae) and in the absence of archival material for re-examination. Lower Cretaceous (possibly even older), diatom-like fossilized remains reported from Korea (ca. 140 Ma; [84]) also carry circular, heterovalvate frustules with highly domed epivalves and flatter hypovalves that are similar to those seen in some Lower Cretaceous diatom resting spores, e.g., *Calyptosporium* [79, 85]. Following these, uncontested, exceptionally well preserved (rather than pyritized) diatoms are known from the Lower Cretaceous (ca.110 Ma, Aptian-Albian; [86]) and show a great diversity of vegetative valve structure. Of a total of 52 species (32 vegetative and 20 spores; [79, 86]), all but two (*Bilingua* and *Kerkis*) have circular, thus non-polar, valves. Similar age diatoms from various other deposits (reviewed in [85, 87]) are generally less species rich and/or well preserved but contain similar frustule designs. The notable exceptions are Lower Cretaceous unusual filaments of cells covered with a continuous, presumably siliceous sheath wrapped around many cells ([85]; figs 36–37 from Queensland, Australia), in a manner similar to modern tube/trichome dwelling organisms. Diatom remains have fossilized in relatively great abundance and diversity since the Upper Cretaceous (~ 85-75Ma, ~300 species, 60 genera; e.g., [29, 30, 88, 89]), at a time when most modern valve architectures and their modern processes were already in evidence.

Molecular clocks, on the other hand, infer a considerably earlier emergence of diatoms, although estimates vary depending on the study. Diatoms are said to emerge “no earlier than 240 Ma” [16, 90]; with the “maximum and minimum divergence time of 250–190 Ma” [91]; or “more likely evolved between 250–183 Ma”, while “less likely between 267–162 Ma” [92]. The latter estimate has since been re-evaluated and even older dates (370–356 Ma) were obtained for the emergence of diatoms [93]. All these estimated oldest dates make diatoms significantly more ancient than the unequivocal fossil record demonstrates. In these timeframes, accepting the earliest estimated diatom emergence (ca. 250 Ma, Permian/Triassic, or ca. 370 Ma Devonian-Carboniferous) would afford diatoms an additional 60–180 million years of evolution (if Liassic *Pyxidicula* proves to be a diatom).

Sims et al. ([16]; p. 366) point out how important it would be to identify which of the lineages of centrics are the most basal among those that have survived to date and then to examine their life histories, if we wish to better understand diversification of diatoms. The two genera whose members are currently known to produce spore-like initial frustules (*Paralia* and *Leptocylindrus*) have been reported among the earliest emerging diatoms [14, 18, 31, 94], depending on taxon sampling and the analysis. In their multigene phylogenies, *Paralia* joins as a sister to the *Hyalodiscus* clade, rooted by *Stephanopyxis* [18] while *Leptocylindrus* roots all the diatoms. Both the *Paralia*-containing clade and that with *Leptocylindrus* received strong support (more than 75% bootstrap and more than 95% posterior probability). The clade comprised of the ancient genera *Stephanopyxis*-*Paralia*-*Hyalodiscus* appears an odd assemblage of taxa when only their fully formed valves are compared [2]. They do however share the same basic silica layer structure with a very regular, quincunx pore pattern (*Stephanopyxis*, *Paralia*, *Hyalodiscus*) known almost unchanged since Upper Cretaceous. These contrast with the simpler valves of *Leptocylindrus*, whose basal silica layer shows only radial ribs and no valve perforations (save a central pore on the valve face of some species), altogether quite similar to the design of incunabular scales on diatom auxospore walls. All this evidence taken together is consistent with the antiquity of the *Leptocylindrus* and *Paralia* lineages and places them in the position where their life histories may indeed inform about the habit of the earliest diatoms. The initial cells of both *Leptocylindrus* [31] and the *Paralia* species shown here are strongly structurally similar to resting spores, and we hypothesize that these diatoms conserved archaic, spore-like morphology in the frustules of their initial cells which we apply here to infer the habit of the early diatoms.

If the above is true, we further hypothesize that following the pre-diatom stages possibly involving the “multiplate diploid spore” (called a "multiplate diploid cyst" in [95]), diatom frustules underwent three phases of evolution. During Phase 1 (possibly Triassic-Jurassic), archaic diatom frustules (both vegetative and initial), might have been spore-like: heterovalvate, relatively poorly perforated and without processes. These archaic frustule-bearing cells (e.g., some possibly similar to frustules of the Korean *Calyptosporium*-like diatom or initial frustules of *Leptocylindrus*) may not have been readily recognizable as belonging to diatoms because they were too similar to “generic” resting spores. These frustules would have been somewhat different from the hypothetical Ur-diatoms in that they were hetero- rather than homovalvate (compare Fig 5J to [95]; fig 16.1d). From some of these spore-like frustule bearing archaic diatoms there evolved more porous vegetative valves without processes. Jurassic specimens of *Phyxidicua* and initial frustules of *P*. *guyana* may reflect such a transition. Thus, in Phase 2, more familiar vegetative valve designs (many homovalvate, chambered and non-chambered; [16]) with various, now extinct valve portulae such as those known from the Lower Cretaceous (ca. 110 Ma) became most common while the sparsely perforated, spore-like frustules might have become limited to the initial cell stage in their life-cycles. In Phase 3, the modern-valve phase of diatom frustule evolution (at least since Upper Cretaceous, but possibly even since Lower Cretaceous, if *Bilingua* and *Kerkis* represent early polar diatoms), modern frustule designs dominate. These contained generally only nearly identical vegetative valves, modern portulae, and most diatoms developed simpler initial cell frustules, more similar to their vegetative counterparts. Spore-like initial frustules remained conserved in a few representatives of the most ancient lineages, such as those containing *Leptocylindrus* and *Paralia*. Should this scenario prove true, then diatom emergence in the Lower Mesozoic suggested by some molecular clocks becomes plausible. This hypothesis can be tested by examination of silica rich sediments in thin section, rather than following chemical dissolution (with strong acids, including HF) which is effective in the recovery of organic material, but is destructive to siliceous remains.

However, whether pre-diatom ancestral stocks originated within haploid, siliceous-scale covered flagellated chrysophycea-like cells [95, 96], filamentous organisms similar to oomycete/xanthophycean-like organisms (wrapped in a sheath with a hexagonal pseudoloculate pattern; [87], p. 133), or one of the straminipilous fungal phyla [16] remains to be determined. The diatom/bolidophyte clade is one of the first photoautotroph divergences from the ancestors they shared with non-pigmented heterokonts in some [93, 97], but not all molecular phylogenies. It is logical to speculate that if the ancestors of the modern “straminipilous fungi” (*sensu* [5]) possessed similarly great diversity of cell-types (both haploid and diploid), cell wall structures, reproductive behavior, and adaptations to a wide range of habitats (parasitic, free living, terrestrial, freshwater or marine, etc.) then a great many new life-histories and life-forms may have been derived from one or another group of these organisms. We observe nonetheless, that extant oomycetes, for example, are diploid in the vegetative state, homothallic, reproduce oogamously, produce large oogonia bearing complex walls with a diameter in excess of the parental hyphae, and produce zygospores following syngamy; characters also seen in non-polar diatoms. On the other hand, silica metabolism and flagellated male gametes are not reported among these “fungi”; although the loss of flagella in modern oomycetes may represent an extreme state of the trend to reduce the flagellar apparatus (expressed in male gametes of centric diatoms) already initiated in the oomycete-diatom last common ancestor. Recent discovery of a great number of “green” genes in diatom genomes has opened the debate on multiple plastid acquisition by the lineage that ultimately diverged into diatoms (reviewed in [91, 98]) and suggests an even more complex evolutionary history, a more diverse range of ancestral traits and possibly also a greater age of these microalgae.

In summary, *Paralia guyana* demonstrates a "heteromorphic" stage in its diploid phase of the life history, previously unknown in diatoms. It consists of resting spore-like zygotes (initial cells) and several valve-types that are structurally intermediate between the spore-like initial and the typical, intercalary vegetative valve morphology. The structural differences between the initial and normal vegetative frustules are so great that it would affect our capacity to recognize them as belonging to the same species, had we not observed their development within auxospores.

Sexual reproductive traits have already contributed insights into deep branching within the diatoms and provided the strongest, biologically relevant context for separation of non-polar from polar diatoms [9, 10, 14], suggesting a closer relationship between the polar centrics and pennates than between non-polar centrics and polar centrics. This view is gaining molecular support with wider taxon sampling and greater number of genes sequenced (compare [21, 23] to [18]). As extant members of the early emerging non-polar centric lineages going back to at least the Upper Cretaceous, *Paralia* and *Leptocylindrus* are in a position to inform on the characters present among the earliest diatoms. Building on our findings presented here, the fossil record [79, 82–85, 88], molecular phylogenies [14, 16, 18] and earlier work on evolution of diatoms by others [95, 96], we suggest three stages in evolution of diatom frustules. Phase 1 involved heterovalvate, non-polar valves and spore-like frustules; phase 2 introduced more perforated, non-polar valves, and less spore-like frustules readily recognizable as diatoms even when modern valve-processes were absent; and phase 3 with homovalvate frustules and modern valve processes. During the latter, polar diatoms (centrics and pennates) diversified into a range of valve outlines facilitated by new structural elements in their auxospore walls allowing for anisodiametric expansion of auxospores leading to the production of polar valves. Some of these diatoms might have lost some of the siliceous structures from the auxospore wall secondarily and thus returned to non-polar valve outlines (e.g. Thalassiosirales).

## Supporting Information

S1 Spreadsheet Source Data. Worksheet “Parent + Initial Cell Diameters” contains original measurements and summary statistics for parent and initial cell diameters, organized by clone and cell type. This data was used to generate Fig 2. Worksheet “Other Metrics” contains original measurements and summary statistics for valve structures reported as means in the text: incunabular scale diameters, copulae slits in 10 μm associated with initial and vegetative valves, and initial cell pores in 10 μm.
(XLS)
